# PAHA study: Psychological Active and Healthy Aging: psychological wellbeing, proactive attitude and happiness effects of whole-body vibration versus Multicomponent Training in aged women: study protocol for a randomized controlled trial

**DOI:** 10.1186/1745-6215-15-177

**Published:** 2014-05-20

**Authors:** Angelo Compare, Cristina Zarbo, Elena Marín, Alessia Meloni, Jacobo A Rubio-Arias, Rosendo Berengüí, Enzo Grossi, Edo Shonin, Gianmaria Martini, Pedro E Alcaraz

**Affiliations:** 1 University of Bergamo, Human Factors and Technologies in Healthcare Research Center, Bergamo, Italy; 2 Universidad Católica San Antonio de Murcia, Campus de los Jerónimos s/n., 30107, Guadalupe, Murcia, Spain; 3 Nottingham Trent University (UK), Nottingham, UK; 4 Villa Santa Maria, Tavernerio, Como. Fondazione Bracco, Milano, Italy; 5 Department of Engineering and Applied Sciences, University of Bergamo, Bergamo, Italy; 6 University of Bergamo, P.le S.Agostino, 2/24129 Bergamo, Italy

**Keywords:** Physical activity, Aging, Women, Quality of life, Prevention, Psychological wellbeing, Proactive attitude, Happiness

## Abstract

**Background:**

Evidence demonstrates that physical exercise and psychological wellbeing are closely interlinked, particularly in older-aged women. However, research investigating how different forms of exercise influence mental health in older-aged women is underdeveloped.

**Methods/Design:**

A randomized controlled trial (N = 300) will assess the relative effectiveness of two different exercise programs (whole-body vibration and Multicomponent Training) for improving psychological wellbeing in older-aged women. The following outcomes will be assessed at three time points (that is, pre, post, and follow-up): psychological wellbeing, proactive attitude, quality of life, and happiness.

**Discussion:**

Results will have important implications for preventing psychological and physiological disease in older-aged women and for managing health-related costs for this population group.

**Trial registration:**

Number NCT01966562 on Clinical Gov database the 8 October 2013

## Background

There were approximately 87 million people aged 65 years old in 2010 in the European Union (17.4% of the total population) compared with just 59.3 million people (12.8%) in 1985 [1]. This phenomenon of an aging population requires a more efficient resources allocation in health care; the demand of health treatments coming from an aging population can be fulfilled through the implementation of new procedures, mostly based on behavioral theories [2]. For instance, the biological aging process is associated with structural and functional deterioration in most physiological systems, including the neuromuscular and cardiovascular systems [3-10]. These deteriorations can affect the ability of older adults to cope with and perform everyday activities. Consequently, there has been a tendency - amongst both the medical community and general public - to view older people as vulnerable, unproductive, and non-contributing members of society. However, in recent years, attitudes have begun to change and emerging research insights demonstrate that an active and engaged lifestyle in older adults contributes not only towards successful aging, but to a healthy economy and society more generally [11]. Indeed, many older adults with active participation in social and leisure activities report good levels of psychological wellbeing [4]. These behavioral approaches to health care are increasingly applied to different treatments.

The activity theory of aging suggests that wellbeing in older adults is promoted by (a) higher levels of participation in social and leisure activities, and (b) role replacement when roles must be relinquished [12]. In addition to the benefits of remaining psychologically stimulated, social and leisure participation may also maintain or promote wellbeing in older adults due to increased physical activity. For example, research demonstrates that the activation of bodily systems promotes a greater range of motion and muscle tone, and improves digestive system functioning and cardiovascular health [11]. More specifically, endurance training is purported to be effective for decreasing fat mass [13], resting heart rate [14] and blood pressure [15], and resistance training can increase basal metabolism [16], bone mineral density (BMD) [17], muscle strength and power [18], and muscle and connective tissue cross-sectional area [19].

A recent review [11] has suggested that successful aging training programs can be distinguished by both the content (that is, the mental and physical demand and/or behavioral aspects) and the social context of the activities. Social activity affects wellbeing by reducing the risks of social isolation and facilitating (a) emotional intimacy, (b) socio-emotional support, (c) reinforcement of self-identity and social roles, and (d) a sense of being valued. Furthermore, productive activity may influence health and wellbeing in older adults through satisfaction with outcomes, economic gains, mental stimulation, comforting personal routines, sense of purpose, and increased self-efficacy and/or self-esteem [20].

As already indicated, psychological wellbeing is a key determinant of quality of life in older people. According to the *Broaden and Build Theory of Positive Emotions*[21], negative emotions serve to narrow focus and attention, whereas positive emotions serve to broaden thoughts and behaviors. Thus, by broadening their thoughts and actions, older-aged adults are better able to cultivate positive emotions and build important coping resources including psychological, intellectual, and physical aptitude. Indeed, there is a strong correlation between quality of life and attitudes towards aging in older adults [22]. Of particular interest in this regard, are gender differences in terms of physical activity engagement. For example, due to factors such as menopausal transition (MT), there is evidence to suggest that older women are less likely to engage in physical activity than older men [23]. This in turn can compound stress levels and increase the risk of depression [24].

Several training methods such as aerobic training, strength training, multicomponent exercise training and whole body vibration (WBV) may improve health outcomes in older-aged women [25]. WBV utilizes high frequency mechanical stimuli (generated by a vibrating platform) that are transmitted through the body where they load the bone and stimulate sensory receptors. Multicomponent exercise training comprises strength training and various balance improving exercises. Significant health improvements are associated with multicomponent exercise training programs in older-adult samples, including the prevention of bone fractures and osteoporosis [22,25,26]. Likewise, WBV is associated with improvements in strength, power, and balance in body composition [27-29] - including in obese women [30]. Findings also demonstrate that occupational exposure to WBV is associated with favorable alterations in mood state that are directly proportional to exposure time [31]. Despite these promising findings, very few studies have focused on the link between these exercise training programs and psychological outcomes (for example, quality of life, psychological wellbeing, happiness, and so on) in older women. Accordingly, the aim of this study is to conduct a randomized controlled trial that assesses these relationships in order to inform the structure and development of exercise training programs for older women.

## Methods/Design

### Hypotheses and design

Starting from current literature, we hypothesize that specific physical activity, WBV, may promote psychological wellbeing and quality of life in female aged subjects, if compared with Multicomponent Training program and normal physical activity. Therefore, we assume an improvement in proactive attitude, quality of life, wellbeing and happiness of WBV training group when compared to and Multicomponent Training and control group.

**Figure 1:**
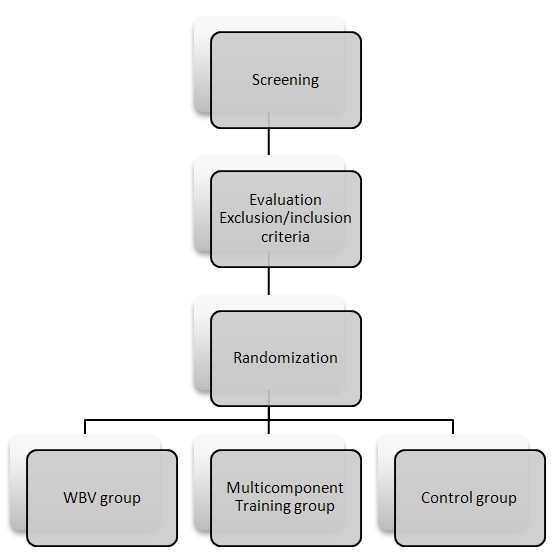
Study design.

The comparison among WBV group, Multicomponent Training group and control group will be assessed in a three-arm randomized controlled clinical trial (Figure 1).

All eligible participants will be approached by trained clinical psychologists and will be screened for psychological aspects concerning the general wellbeing, the proactive attitude and the health status. Participants will, moreover, be informed of the protocol and procedures prior to their involvement and written consent to participate will be obtained. Informed consent will be sought for: (a) using anonymous data from the questionnaires for reports and scientific publications; and (b) for informing the general practitioner (GP) about the study results.

### Setting

The study will be conducted in collaboration with the University of Bergamo (Italy) and the Catholic University of San Antonio of Murcia (Spain). The Psychological scale administrations and training intervention will be conducted at the biomechanics lab in the UCAM Research Center for High Performance Sports. Different settings will be utilized due to the different nature of the exercises. The WBV training will in fact be carried out at the lab and the Multicomponent Training in a garden close to the research center.

### Subjects

A non-probability sampling intentional/convenience will be used for sample selection. Participants with the following eligibility criteria will be included in the present study: women; age ranged between 55 to 75 years old. Exclusion criteria included: male sex; age lower than 55 years old; present levels of bone mineral density (BMD) lower than 70 g/cm^2^; being treated for a disease that can affect bone structure or neuromuscular system; have orthopedic prosthetic implants in the lower limbs and/or spine; have herniated discs; suffer ocular diseases that affect the retina; suffer severe cardiovascular diseases; have a pacemaker, or osteosynthesis material *in situ*; severe mental illness (active psychosis/suicide risk/severe dementia); linguistic limitations (such as stuttering/untreated audio impairment); a significant functional problem (such as unconsciousness/connection to respiration device/confinement to a wheelchair or bed/severe walking disability/need of help with basic daily activities), major depression, anxiety according to *Diagnostic and Statistical Manual of Mental disorders, version 4* (DSM-IV) criteria.

### Randomization

Randomization will be performed by statistical experts of the team study using a computerized random number generator at (http://www.randomizer.org/). To obtain equal numbers in both conditions, a block randomization design is chosen. After the completion of the inclusion procedure, the clinical psychologist will obtain the file containing the condition to which the patient is allocated.

### Interventions

The interventions protocol will consist of six months of training for both groups of vibratory platform and Multicomponent Training. Both groups, WBV and Multicomponent Training, will follow three training sessions per week, with a recovery time between sessions ranging from 24 to 72 hours.

### WBV training

After the warm-up consisting in eight minutes of static cycling, active stretching and joint mobility, participants will perform the WBV training. The vibration stimulus will consist of uniform vertical oscillations Power Plate® Next Generation (Power Plate North America, Northbrook, IL, USA). Subjects will stand on the platform holding quarter squat positions with the feet shoulder-width apart. Then, they will perform ankle extensions with the following work sequence (establishing a rhythm of 100 bpm: 1 bpm for the concentric phase; and 5 bpm for the eccentric phase). After the familiarization 2-weeks, subjects will train 3 days per week for 6 months (72 sessions) using a vibrating training program that will begin with 5 sets and a frequency of 35 Hz per session and increasing by 11 sets and 40 Hz frequency for the last month, maintaining a series of parameters: vibration amplitude (4 mm) working time (60 seconds) and recovery time (60 seconds).

### Multicomponent Training

In the same way, and after the warm-up consisting in eight minutes of static cycling, active stretching and joint mobility, participants will perform the Multicomponent Training. This training will combine vertical jumps and high-intensity walking. During the first month, small reactive vertical jumps (without knee and ankle flexion) will be performed. After the first month, subjects will perform drop jumps progressively starting at a height of 5 cm and finishing at 25 cm at the end of the program (increases of 5 cm each month). Additionally, the sets will be increased from 4×10 jumps to 6×10 each week, finishing the last week with 4×10. In this sense, the drop jumps will be the same each month but the total load (imposed by height) will increase progressively. Regarding the aerobic exercise, the load will increase progressively along the six months. The intensity will range between 50 to 75% of reserve heart rate; the volume will range between 30 to 60 minutes.

### Control group

Control group participants will be asked to continue their daily lives normally and maintain regular physical activity during the six-month duration of the study.

### Measurements

Besides the psychological screening baseline at one week after the beginning of training (T1), all participants will be assessed at three months (T2) and six months (T3).Moreover, at the baseline assessment, questions on demographic variables (for example, age, marital status, work, educational level, socioeconomic status), psychiatric history (for example, previous diagnosis of depression and/or anxiety, family history regarding psychiatric diagnosis), and/or health behaviors (for example, alcohol use, smoking habits, physical activity) will be included.

All participating sample were screened for psychological aspects by the following standardized measures:

#### *Primary outcome measures*

**SF-12 Health Survey** It is a self-administered test that assesses the global health status by the subjective point of view of the subject. It has been divided into two principal subscales: the Physical Component Summary (PCS) and the Mental Component Summary (MCS). In particular, it allows the assessment of concepts concerning health, physical functions, pain, general health, vitality, social functioning, emotional functioning and mental health [32].

#### The Psychological General Well-Being Index (PGWBI)

It is a self-administered test with 22 items that assesses the subjective sensation of psychological wellbeing. It has been divided intosix categories: anxiety, depression, self-control, positivity and wellbeing, health and vitality [33].

### *Secondary outcome measures*

**Subjective Happiness Scale (SHS)** It is a four-item scale for assessing subjective happiness. Two items ask respondents to characterize themselves using both absolute ratings and ratings relative to peers, whereas the other two items offer brief descriptions of happy and unhappy individuals and ask respondents the extent to which each characterization describes them. The SHS has been validated in 14 studies with a total of 2,732 participants. Preliminary results have indicated that the SHS has high internal consistency, which has been found to be stable across samples. Test-retest and self-peer correlations have suggested good to excellent reliability, and construct validation studies of convergent and discriminant validity have confirmed the use of this scale to measure the construct of subjective happiness [34].

### 

**Proactive Attitude Scale (PA)** It assesses the presence of a proactive attitude that is a relatively persistent personal belief in the rich potential of changes that can be made to improve oneself and one’s environment. The proactive attitude has implications for motivation and action. This includes various facets such as resourcefulness, responsibility, values and vision. The psychological construct of Proactive Attitude (PA) presents a correlation of r = .56 with general self-efficacy [35].

## Ethical principles

The study of this protocol will not involve the administration of drugs. However, the investigator will be responsible for ensuring that the study will be conducted in accordance with the principles defined by the 18th World Medical Assembly (Helsinki, 1964) and subsequent amendments established by the 29th (Tokyo, 1975), 35th (Venice, 1983), 41st (Hong Kong, 1989), and the 48th World Medical Assembly (Somerset West, South Africa, 1996), and 52nd (Edinburgh, Scotland, 2000) General Assembly. The study will also be conducted in accordance to the Ministerial Decree of 15 July 1997 transposing the text of the rules of Good Clinical Practice for human trials of medical products within the EEC. At the time this manuscript was submitted, full approval by the Human Subjects Ethics Committee of the Universidad Católica San Antonio de Murcia had been obtained.

## Planned statistical analyses

The demographic and clinical characteristics of the three study groups will be compared at baseline to verify their homogeneity. To do this, analysis of variance (ANOVA) for continuous variables and chi-square test of Mantel-Haenszel test for discrete variables will be used. Complete and intent-to-treat analysis will be accomplished to compare primary outcome treatments. First, prevalence of happiness, proactive attitude, psychological wellbeing and quality of life will be displayed infrequency tables and both conditions (control and intervention)will be compared using the chi-square (Fisher’s exact test when appropriate) and Student’ s *t*-test, for discrete and continues variables respectively. The effect of the intervention will be determined using multilevel analyses (mixed effect regression models) to compare baseline and follow-up measures of all continuous data. In all these analyses a *P*-value of <0.05 will be considered statistically significant.

## Sample size calculation

In this study, the change in scores in PGWBI and SF-12 are chosen as primary outcome measures to determine the effect of whole-body vibration approach. A difference of 0.5 standard deviations is considered necessary to find a clinically significant effect of the intervention. In order to detect this difference and assuming an 80% power, a minimum of 80 patients is needed in each outcome measure [36]. When assuming that 20% of participants dropout (quite a normal figure in this type of research), a minimum of 23 subjects per condition is needed to maintain sufficient power. We expect that in order to reach such a number, anticipating a response rate of 70% of which 20% is eligible, a total of 300 aged women will need to be screened.

## Discussion

This article describes the background, objectives and design of a randomized controlled trial that will test the role of specific physical activity, WBV and Multi-component training, on the promotion of psychological wellbeing, proactive attitude and happiness in aging women. Although several studies have been conducted on the aging status and prevention, very few randomized controlled trials have focused on the link between specific physical activity and psychological wellbeing in adult women. A randomized controlled trial that assesses their relationships may be useful in order to improve the quality of prevention programs and then promote the general wellbeing of the national population. Given the progressive increase of the elderly population in Europe, prevention plays a key role in containing health costs and comprises a large portion of health expenditure. Because of the known link between lifestyle and disease in aging and the high cost associated with lifestyle diseases, improving and promoting health in middle and older age is in fact becoming a European health and research priority. The comprehensive cost-benefit analysis of these behavioral prevention treatments, to determine estimates of the health care cost savings obtainable through these approaches, is left for future research.

## Trial status

PAHA Protocol has been registered on Clinical Gov database with ID NCT01966562. The present study trial was conceived and designed in 2012.

## Abbreviations

BMD: bone mineral density; DSM-IV: *Diagnostic and Statistical Manual of Mental disorders, version 4*; MCS: Mental Component Summary; MT: menopausal transition; PA: proactive attitude; PCS: Physical Component Summary; PGWBI: The Psychological General Well-Being Index; SHS: Subjective Happiness Scale; WBV: whole-body vibration.

## Competing interests

The authors declare that they have no competing interests.

## Authors’ contributions

AC conceived of the study, and participated in its design and drafted the manuscript. AP, ME, RJ, MG, EG, SE and RA performed the statistical analysis. ZC and MA participated in the design of the study and helped to draft the manuscript. All authors have been involved in writing this manuscript and have approved the final manuscript and its submission.
